# Vitrectomy in Small idiopathic MAcuLar hoLe (SMALL) study: Internal limiting membrane peeling versus no peeling

**DOI:** 10.1111/aos.16778

**Published:** 2024-10-14

**Authors:** Paola Marolo, Paolo Caselgrandi, Matteo Fallico, Guglielmo Parisi, Enrico Borrelli, Federico Ricardi, Francesco Gelormini, Luca Ceroni, Michele Reibaldi, SMALL Study Group, Tommaso Micelli Ferrari, Massimo Lorusso, Vito Primavera, Gianluigi Giuliani, Cesare Mariotti, Marco Lupidi, Luca Ventre, Giulia Pintore, Lorenzo Motta, Matteo Ripa, Francesco Boscia, Giacomo Boscia, Mario R Romano, Mariantonia Ferrara, Kacerik Miroslav, Daniele Marchina, Barbara Parolini, Enrico Peiretti, Viola Marchiori, Roberto Dell’Omo, Marzia Affatato, Teresio Avitabile, Andrea Russo, Antonio Longo, Vincenzo Scorcia, Adriano Carnevali, Rodolfo Mastropasqua, Lisa Toto, Agostino Salvatore Vaiano, Riccardo Merli, Marco Mura, Marco Pellegrini, Fabrizio Giansanti, Cristina Nicolosi, Matteo Badino, Nicola Pallozzi Lavorante, Maria T. Sandinha, Francesco Maria D’Alterio, Mario Damiano Toro, Robert Rejdak, Paolo Chelazzi, Claudia Azzolini, Francesco Viola, Caterina Donà, Matteo Giuseppe Cereda, Marco Casaluci, Marco Codenotti, Lorenzo Iuliano, Grazia Pertile, Daniele Sindaco, Stefano De Cillà, Andrea Muraca, Vincenza M.E. Bonfiglio, Maria Vadalà, Alberto La Mantia, Viviana Randazzo, Tito Fiore, Gianluigi Tosi, Rino Frisina, Chiara Angeli, Marco Coassin, Mariateresa Laborante, Tommaso Rossi, Pamela Cosimi, Stanislao Rizzo, Matteo Mario Carlà, Magda Gharbiya, Giuseppe Maria Albanese, Luigi Caretti, Edoardo Angelini, Gian Marco Tosi, Tommaso Bacci, David H Steel, Nikolaos Dervenis, Iordanis Vagiakis, Daniele Tognetto, Marco Rocco Pastore, Francesco Faraldi, Carlo Alessandro Lavia, Paolo Lanzetta, Daniele Veritti, Leopoldo Rubinato, Paolo Radice, Andrea Govetto

**Affiliations:** ^1^ Department of Surgical Sciences, Section of Ophthalmology University of Turin Turin Italy; ^2^ Department of Ophthalmology University of Catania Catania Italy; ^3^ University of Turin Turin Italy

**Keywords:** closure rate, macular hole, no‐peeling, optical coherence tomography, peeling, small, visual acuity, vitrectomy, vitreomacular traction

## Abstract

**Purpose:**

To compare vitrectomy with and without internal limiting membrane (ILM) peeling in small idiopathic macular holes.

**Methods:**

Retrospective multicentre study including consecutive eyes with ≤250 μm idiopathic macular hole treated with vitrectomy. The primary outcome was hole closure rate. Best‐corrected visual acuity (BCVA) change, closure patterns on optical coherence tomography, rates of external limiting membrane (ELM) and ellipsoid zone (EZ) recovery, and rate of complications were also investigated.

**Results:**

In total, 693 eyes were included. Hole closure rate was 98% in the peeling and 85% in the no‐peeling group (*p* < 0.001). At 12 months, mean BCVA change was 0.38 ± 0.22 logMAR in the peeling and 0.45 ± 0.21 logMAR in the no‐peeling group (*p* = 0.02); 66% versus 80% of eyes had a U‐shaped morphology, respectively; EZ recovery rate was 75% and 93%, respectively (*p* = 0.02). In the no‐peeling group, eyes with a vitreomacular traction (VMT) showed a 96% closure rate, comparable to the peeling group (*p* = 0.40). The incidence of adverse events was similar except for dissociated optic nerve fibre layer (55% in the peeling vs. 9% in the no‐peeling group, *p* < 0.001).

**Conclusions:**

In small idiopathic macular holes, ILM peeling provides a higher closure rate compared to no‐peeling; however, if a VMT is present closure rates are comparable. In closed macular holes, the no‐peeling technique provides advantages in terms of visual outcome and anatomical recovery.

## 1 INTRODUCTION

Idiopathic macular hole (MH) is a cause of severe visual impairment featuring central visual loss and metamorphopsia. The treatment of idiopathic MH aims at hole closure and functional improvement. Despite a high anatomic success rate, functional results appear variable (Essex et al., 2018; Jackson et al., 2013). Anatomical and functional outcomes have been shown to be better for MHs with a small size, with a higher chance of achieving an optimal recovery of visual function (Fallico et al., 2021).

Currently, conventional treatment for small and medium idiopathic MHs involves vitrectomy with internal limiting membrane (ILM) peeling and gas tamponade. The introduction of ILM peeling was a milestone in MH surgery, being considered a key step for hole closure. However, peeling manoeuvres might be associated with drawbacks such as dye‐related toxicity and mechanical injury to retinal layers, which could limit a full restoration of visual function (Gelman et al., 2015).

The International Vitreomacular Traction Study (IVTS) Group classified as ‘small’ those MHs with a ≤ 250 μm aperture size (Duker et al., 2013). The cut‐off for small holes was set at 250 μm because MHs of 250 μm or smaller have a good response to pharmacologic vitreolysis (Duker et al., 2013; Stalmans et al., 2012). Small idiopathic MHs have been shown to be responsive to pneumatic vitreolysis as well. This treatment has shown a closure rate as high as 66% of cases for small MHs associated with vitreomacular traction (VMT) (Chan et al., 2017; Chan et al., 2021). The efficacy of both pharmacologic and pneumatic vitreolysis suggested that anatomical closure of small MHs can be achieved without performing an ILM peeling.

To date, no studies have compared vitrectomy with ILM peeling versus vitrectomy without ILM peeling in small MHs according to the new IVTS classification. On this basis, the purpose of our study was to investigate anatomical and functional outcomes of vitrectomy with and without ILM peeling for small idiopathic MHs and to assess which variables might influence surgical outcomes.

## 2 MATERIALS AND METHODS

This multicentre, retrospective cohort study included consecutive eyes with small idiopathic MHs treated with primary pars plana vitrectomy between January 2019 and June 2022 at 42 vitreoretinal Units in Europe: Molinette Hospital, University of Turin, Italy; Miulli Hospital, Acquaviva delle Fonti, Bari, Italy; Della Murgia Fabio Perinei Hospital, Altamura, Italy; Riuniti Hospital, University of Ancona, Italy; Beauregard Hospital, Aosta, Italy; William Harvey Hospital, East Kent Hospitals University, NHS Foundation Trust, Ashford, UK; Giovanni XXIII Hospital, Aldo Moro University, Bari, Italy; Gavazzeni‐Castelli Hospital, Humanitas University, Bergamo, Italy; Papa Giovanni XXIII Hospital, Bergamo, Italy; San Giovanni di Dio Hospital, University of Cagliari, Italy; Antonio Cardarelli Hospital, University of Molise, Campobasso, Italy; Gaspare Rodocolico‐San Marco University Hospital, Catania, Italy; Mater Domini Hospital, Magna Graecia University, Catanzaro, Italy; Santissima Annunziata Hospital, Gabriele D'Annunzio University of Chieti‐Pescara, Chieti, Italy; Santa Croce e Carle Hospital, Cuneo, Italy; St. Anna Hospital, University of Ferrara, Italy; Careggi University Hospital, Florence, Italy; San Martino Hospital, Genova, Italy; St. Paul's Eye Unit, The Royal Liverpool University Hospital, Liverpool, UK; Medical University of Lublin, Poland; Istituto Clinico Città Studi, Milan, Italy; Fondazione IRCSS Ca′ Granda Ospedale Maggiore Policlinico, University of Milan, Italy; Sacco Hospital, Milan, Italy; San Raffaele Scientific Institute, Vita‐Salute University, Milan, Italy; IRCCS Sacro Cuore Don Calabria, Negrar di Valpolicella, Italy; Maggiore della Carità Hospital, Piemonte Orientale University, Novara, Italy; Paolo Giaccone Hospital, University of Palermo, Italy; Riuniti Villa Sofia‐Cervello Hospital, Palermo, Italy; S.Maria della Misericordia Hospital, University of Perugia, Italy; Guglielmo da Saliceto Hospital, Piacenza, Italy; Campus Bio‐Medico University, Rome, Italy; Fondazione Bietti, Rome, Italy; Fondazione Policlinico Universitario Gemelli, Sacro Cuore Catholic University, Rome, Italy; Policlinico Umberto I Hospital, Sapienza University, Rome, Italy; Santa Maria della Misericordia Hospital, Rovigo, Italy; Le Scotte University Hospital, Siena, Italy; Sunderland Eye Infirmary, Sunderland, UK; AHEPA Hospital, University of Thessaloniki, Greece; Riuniti Hospital, University of Trieste, Italy; Mauriziano Hospital, Turin, Italy; University of Udine, Italy; and Circolo e Fondazione Macchi Hospital, Varese, Italy.

The study protocol was in agreement with the tenets of the Declaration of Helsinki and was approved by Institutional Review Board at each participating site, unless the collection of anonymized data was deemed as part of a service evaluation or audit. The following eligibility criteria were applied: (a) idiopathic full thickness small MH, as defined by the IVTS Group (Duker et al., 2013), with an aperture size ≤250 μm treated with primary vitrectomy; (b) pseudophakic status at the time of surgery or phakic eye undergoing combined phacovitrectomy; (c) follow‐up ≥6 months; (d) preoperative and postoperative spectral domain optical coherence tomography (sd‐OCT) imaging. The following exclusion criteria were considered: high myopia (>6 diopters or axial length >25.5 mm); MH secondary to other conditions or traumatic hole; glaucoma; amblyopia; other eye diseases that could influence visual function; any previous intraocular surgery except uneventful cataract surgery; use of silicone oil as tamponade; and poor quality OCT imaging.

All patients received a complete eye examination preoperatively and post‐operatively during the follow‐up period. Best‐corrected visual acuity (BCVA) was measured using the Early Treatment Diabetic Retinopathy Study (ETDRS) charts and then converted into logarithm of minimum angle (logMAR) values. Intraocular pressure (IOP) measurement, slit lamp examination and dilated fundus examination were also performed. MH size was defined as the minimum linear diameter (MLD) on OCT imaging, which was the diameter at the narrowest point between the hole edges through the fovea centre (Ch'ng et al., 2018). The OCT basal hole diameter (BHD) was defined as the diameter between hole edges at the retinal pigment epithelium level.

A standard pars plana vitrectomy was performed, combined with phacoemulsification and intraocular lens implantation in all phakic eyes. The ILM was stained using either a blue or green dye. The blue dyes included 0.025% brilliant blue G, 0.15% trypan blue or a combination of both dyes, while the green dye used in only 6 eyes was indocyanine green at a concentration of 0.1 mg/mL. Regarding the preparation and dilution of the indocyanine green, 25 mg of the dye was dissolved in 10 mL of distilled water to obtain a concentration of 2.5 mg/mL. From this, 1 mL was diluted with 4 mL of BSS Plus to achieve a concentration of 0.5 mg/mL. Then, 1 mL of this solution was further diluted with 4 mL of BSS Plus to reach a final concentration of 0.1 mg/mL. A volume of 0.2 mL of this 0.1 mg/mL indocyanine green dye was injected to stain the ILM and washed out within 10 s. The decision to perform conventional ILM peeling or not was based on the surgeon's discretion. When conventional ILM peeling was performed, the extent of peeling was within two‐disc diameters from the centre of the fovea. When the inverted flap technique was adopted, the ILM was peeled circumferentially within two‐disc diameters from the fovea centre, leaving it attached to the edges of the macular hole. The flap was trimmed, inverted and left to cover the macular hole with no attempt to insert the flap into the hole. Perfluorocarbon liquids and/or viscoelastic devices were not used to flatten or stabilize the flap in any case. In case of any deviation from inverted flap protocol, eyes were excluded from the study. At the end of the procedure, the vitreous cavity was left filled with air or gas, including 20% sulfur hexafluoride (SF_6_), 12% pctafluoropropane (C_3_F_8_) or 16% perfluoroethane (C_2_F_6_). A face‐down position was recommended for 3–5 days.

At each centre, two independent and experienced investigators reviewed patients charts and collected preoperative, intraoperative and postoperative data. In case of disagreement, a third investigator was involved to achieve consensus. Preoperative data included demographic data; lens status; BCVA; OCT parameters, such as MLD, BHD, the presence of epiretinal membrane (ERM), the presence of VMT (Duker et al., 2013) and the presence of epiretinal proliferation. MH associated epiretinal proliferation was identified on OCT scan as a homogenous medium reflectivity tissue located on epiretinal surfaces. Intraoperative data included vitrectomy calliper (23, 25 or 27 gauge); type of surgery (vitrectomy or combined phacovitrectomy); information on whether ILM peeling was performed and which type of peeling (conventional peeling or inverted flap); type of intraoperative dye (none, blue or green); and type of tamponade (gas or air). Postoperative data included MH closure; BCVA; OCT parameters, such as type of MH closure (U‐shape, V‐shape, W‐shape (Imai et al., 1999)), external limiting membrane (ELM) and ellipsoid zone (EZ) status; rate of adverse intraoperative and postoperative complications including retinal tear, endophthalmitis, cystoid macular oedema, vitreous haemorrhage, retinal detachment, dissociated optic nerve fibre layer (DONFL) and swelling of the arcuate retinal nerve fibre layer (SANFL).

Postoperative visits were classified as 1‐month follow‐up (30 ± 7 days), 6‐month follow‐up (6 months±15 days) and 1‐year follow‐up (12 ± 1 months).

At the first follow‐up (1 month), all eyes were included. All the analysis after 1 month included only eyes with MH closure, whereas the data of eyes with open MH were collected but not included in the analysis.

According to whether the ILM peeling was performed or not, included eyes were divided into two groups, namely the peeling group and the no‐peeling group.

The primary outcome of this study was to compare MH closure rate and postoperative visual change between the two groups. Type of MH closure, ELM and EZ integrity, and rate of complications were investigated as secondary outcomes. The influence of clinical and surgical variables on anatomical outcome was considered as a secondary outcome as well.

### 2.1 Statistical analysis

Descriptive statistics were reported as mean and standard deviation for continuous variables or frequency and percentage for qualitative variables. The Shapiro–Wilk test was used to evaluate the normal distribution of continuous variables. Differences between two groups were explored by using the Mann–Whitney *U* and the Student *t*‐tests for nonparametric and parametric variables, respectively. Categorical variables were tested by using the chi‐square or, when needed, the Fisher exact tests. Differences between continuous variables in more than two groups were assessed with the Wilcoxon signed‐rank test and ANOVA for nonparametric and parametric variables, respectively. Mean visual change in each group was calculated throughout the follow‐up. Postoperative mean visual change and closure rate were compared between the two groups by using the Mann–Whitney *U* and chi‐square tests, respectively. In each group, mean values of visual acuity detected at different time‐points were compared by using the ANOVA test. The chi‐square was used to explore differences in closure type between the two groups (i.e. rates of U‐shape, V‐shape and W‐shape). The chi‐square or, when needed, Fisher exact tests were used to explore differences in ELM and EZ recovery rates and adverse events rates. Binary logistic regression was applied to evaluate the dependence of one or more independent variables on a dichotomous variable, and each independent variable was tested with a univariate analysis. Variables showing a *p* value ≤0.2 on the univariate analysis were investigated by multivariate logistic regression analysis. Odds ratio with 95% confidence interval (CI) was calculated. Exponentiated beta coefficient (Exp[B]) represented the odds ratio, indicating the change in odds for a one‐unit increase in the predictor variable.

A *p* value ≤0.05 was considered statistically significant. Analyses were performed using IBM SPSS Statistics (Version 21.0; IBM Corp).

## 3 RESULTS

Six hundred ninety‐three eyes of 693 patients (mean age 69 ± 8 years) with at least 6 months of follow‐up were included in the study. Among these, 639 eyes received ILM peeling and 54 eyes did not. Baseline demographic, clinical and surgical characteristics of enrolled patients divided by type of surgical procedure (peeling group vs. no‐peeling group) are shown in Table 1.

**TABLE 1:** Baseline characteristics of the study population.

Characteristic	Peeling (*N* = 639)	No‐peeling (*N* = 54)	*p* value
Women, *n* (%)	337 (53)	37 (69)	0.025
Age, mean (±SD)	69 (±8)	70 (±8)	0.4
Diabetes, *n* (%)	59 (10)	7 (13)	0.4
Lens status, *n* (%)			
Pseudophakic	306 (48)	28 (52)	>0.9
BCVA (logMAR), mean (±SD)	0.55 (±0.25)	0.47 (±0.25)	0.018
MLD (𝜇m), mean (±SD)	183 (±55)	171 (±52)	0.034
BHD (𝜇m), mean (±SD)	489 (±241)	390 (±198)	0.001
ERM, *n* (%)	240 (38)	1 (2)	<0.001
VMT, *n* (%)	190 (30)	23 (43)	0.018
Epiretinal proliferation, *n* (%)	155 (25)	2 (4)	<0.001
Vitrectomy calliper, *n* (%)			0.7
23 G	156 (24)	11 (20)	
25 G	398 (62)	34 (63)	
27 G	85 (13)	9 (17)	
Combined phacovitrectomy, *n* (%)	333 (52)	26 (48)	0.5
Type of peeling, *n* (%)			<0.001
Conventional peeling	389 (61)	0 (0)	
Inverted flap	250 (39)	0 (0)	
Type of dye, *n* (%)			<0.001
None	3 (1)	42 (78)	
Blue	630 (99)	12 (22)	
Brilliant blue G	460 (72)	12 (22)	
Brilliant blue G + trypan blue	170 (27)	0 (0)	
Indocyanine green	6 (1)	0 (0)	
Type of tamponade, *n* (%)			0.001
Air	65 (10)	0 (0)	
Gas	574 (90)	54 (100)	
SF_6_	446 (70)	48 (89)	
C_3_F_8_	99 (16)	6 (11)	
C_2_F_6_	29 (5)	0 (0)	

Abbreviations: BCVA, best‐corrected visual acuity (logMAR [logarithm of the minimum angle of resolution]); BHD, basal hole diameter; C_2_F_6_, perfluoroethane; C_3_F_8_, octafluoropropane; ERM, epiretinal membrane; MLD, minimum linear diameter; SD, standard deviation; SF_6_, sulfur hexafluoride; VMT, vitreomacular traction.

### 3.1 Macular hole closure rate

Overall, 627 eyes out of 639 in the peeling group and 46 eyes out of 54 in the no‐peeling group achieved primary MH closure. The primary MH closure rate was significantly higher in the peeling group compared with the no‐peeling group (98% versus 85%; odds ratio [OR] 7.89; 95% CI, 3.371–18.46; chi‐square, *p* < 0.001).

All eyes not achieving MH closure after the first vitrectomy underwent a second vitrectomy with different techniques. These eyes were excluded from subsequent analysis. The MH closure rate after second surgery was 100%.

### 3.2 Visual outcomes

Mean BCVA significantly improved in both groups from baseline during follow‐up (ANOVA, *p* < 0.001). Mean postoperative BCVA change from baseline was not statistically different between the two groups at 1 month, whereas there was a higher mean BCVA change at 6 and 12 months in the no‐peeling group (Mann–Whitney, *p* = 0.04 at 6 months, and *p* = 0.02 at 12 months) (Table 2).

**TABLE 2:** Mean BCVA changes from baseline in the peeling versus the no‐peeling group.

∆BCVA (logMAR), Mean (±SD)	Peeling	No‐peeling	*p* value
1 month	0.27 (±0.23) (*N* = 639)	0.26 (±0.26) (*N* = 54)	0.2
6 months	0.34 (±0.26) (*N* = 622)	0.40 (±0.24) (*N* = 46)	0.04
12 months	0.38 (±0.22) (*N* = 501)	0.45 (±0.21) (*N* = 38)	0.02

Abbreviations: BCVA, best‐corrected visual acuity (logMAR [logarithm of the minimum angle of resolution]); SD, standard deviation.

**FIGURE 1:**
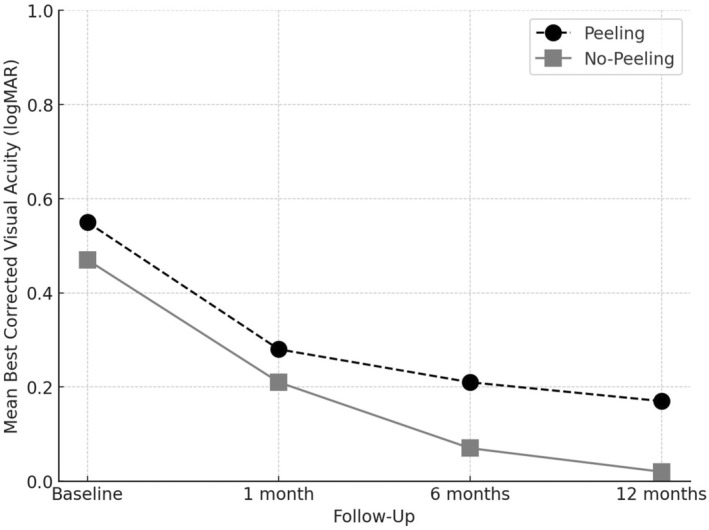
Mean best‐corrected visual acuity (BCVA) over time for peeling versus no‐peeling group. BCVA was assessed at baseline, 1 month, 6 months and 12 months after treatment. The peeling group (black circles, dashed line) showed a consistent improvement, with BCVA decreasing from 0.55 logMAR at baseline to 0.17 logMAR at 12 months (ANOVA, *p* < 0.001). The no‐peeling group (grey squares, solid line) also demonstrated a significant improvement, with BCVA decreasing from 0.47 logMAR at baseline to 0.02 logMAR at 12 months (ANOVA, *p* < 0.001).

Mean BCVA was statistically different between the peeling group and the no‐peeling group at baseline, 6 and 12 months, respectively, 0.55 ± 0.25 logMAR and 0.47 ± 0.25 logMAR (Mann–Whitney, *p* = 0.018), 0.21 ± 0.20 logMAR and 0.07 ± 0.16 logMAR (Mann–Whitney, *p* = 0.046) and 0.17 ± 0.19 logMAR and 0.02 ± 0.17 logMAR (Mann–Whitney, *p* = 0.014). Differently, there was no statistically significant difference between the two groups at 1 month, respectively, 0.28 ± 0.23 logMAR in the peeling group and 0.21 ± 0.18 logMAR in the no‐peeling group (Mann–Whitney, *p* = 0.2) (Figure 1).

### 3.3 OCT anatomical outcomes

The type of closure morphology and integrity of outer retinal layers at 12 months are shown in Table 3.

**TABLE 3:** Closure‐related outcomes in the peeling versus the no‐peeling group at 12 months.

Outcome	Peeling (*N* = 501)	No‐peeling (*N* = 38)	*p* value
Closure morphology, *n* (%)			0.001
U shape	331 (66%)	30 (80%)	–
V shape	60 (12%)	8 (20%)	–
W shape	110 (22%)	0 (0%)	–
ELM recover, *n* (%)	451 (90%)	37 (98%)	0.1
EZ recover, *n* (%)	376 (75%)	35 (93%)	0.02

Abbreviations: ELM, external limiting membrane; EZ, ellipsoid zone.

The distribution of closure morphologies at 12 months was statistically different between the two groups (chi‐square, *p* = 0.038). The rate of U‐shaped and V‐shaped closure type was higher in the no‐peeling group, while the rate of W shaped closure type was higher in the peeling group.

At 12 months, the rate of ELM integrity was not different in the peeling group compared with the no‐peeling group, whereas the rate of EZ integrity was lower in the peeling group compared with the no‐peeling group (chi‐square, *p* = 0.1 and *p* = 0.02, respectively).

### 3.4 Multiple regression analysis

Table 4 summarizes the results of the multiple binary logistic regression analysis in the peeling group and the no‐peeling group.

**TABLE 4:** Results for multiple regression analysis with macular hole closure as dependent variable.

	Exp(B)	*p* value
Peeling		
Baseline BCVA	0.181	0.125
Presence of epiretinal membrane	0.247	0.043
Lens status (pseudophakia)	0.474	0.226
Type of tamponade (gas)	4.043	0.031
No‐peeling		
Age	0.867	0.097
Presence of VMT	10.626	0.048
Lens status (pseudophakia)	1.492	0.724

Abbreviations: BCVA, best‐corrected visual acuity; Exp(B), exponentiated beta coefficient; VMT, vitreomacular traction.

Multiple regression analysis with MH closure set as a dependent variable showed that the presence of an ERM at baseline decreased the probability of MH closure (Exp[B] = 0.247, *p* = 0.043), whereas the choice of gas tamponade during surgery increased the probability of MH closure (Exp[B] = 4.043, *p* = 0.031) in the peeling group. In the no‐peeling group, the presence of a VMT at baseline increased the probability of MH closure (Exp[B] = 10.626, *p* = 0.048).

### 3.5 Subgroup MH closure rate

A subgroup analysis was conducted on eyes of the no‐peeling group that presented with a VMT at baseline.

Overall, 22 eyes out of 23 with a VMT at baseline achieved primary MH closure.

The primary MH closure rate of eyes with VMT at baseline in the no‐peeling group was not statistically different from the primary MH closure rate of eyes in the peeling group (respectively, 96% vs. 98%, chi‐square, *p* = 0.40).

### 3.6 Adverse events

The incidence of intraoperative and postoperative adverse events was similar between the two groups with the exception of DONFL appearance (55% in the peeling group vs. 9% in the no‐peeling group, chi‐square, *p* < 0.001) (Table 5).

**TABLE 5:** Intraoperative and postoperative adverse events.

Adverse event	Peeling (*N* = 639) No of events (%)	No‐peeling (*N* = 54) No of events (%)	*p* value
Retinal tear	11 (1.7%)	1 (1.9%)	>0.9
Endophthalmitis	0 (0%)	0 (0%)	–
Cystoid macular oedema (12 months)	12 (1.9%)	0 (0%)	–
Vitreous haemorrhage	15 (2.3%)	1 (1.9%)	0.8
Retinal detachment	5 (0.8%)	0 (0%)	–
DONFL	351 (55%)	5 (9%)	<0.001
SANFL	34 (5.3%)	0 (0%)	–

Abbreviations: DONFL, dissociated optic nerve fibre layer; SANFL, swelling of the arcuate nerve fibre layer.

## 4 DISCUSSION

The Small idiopathic MAcuLar hoLe (SMALL) study was designed to collect real‐life evidence on surgical outcomes of small idiopathic MHs. Report 1 compared outcomes of vitrectomy with and without ILM peeling, showing a higher closure rate when vitrectomy was associated with ILM peeling. However, the closure rate was comparable between the two techniques in eyes with a VMT associated with MH. The no‐peeling technique provided better visual outcomes, with a higher chance of achieving a normal foveal morphology and full integrity of photoreceptor layers.

The study included 693 eyes from 42 European Ophthalmologic Centers, providing a reliable picture as to how small MHs have been treated in the last few years.

Less than 10% of small MHs received a no‐ILM peeling vitrectomy. This demonstrated that ILM peeling is the current treatment of choice for small MHs.

Vitrectomy with ILM peeling has been shown to yield excellent closure rates in small‐to‐medium MHs ranging from 85% to 100% (Marques et al., 2020; Rahimy & McCannel, 2016). Closure rates of no‐ILM peeling vitrectomy have been reported between 50% and 100% (Lois et al., 2011; Tadayoni et al., 2006; Tognetto et al., 2006) for MHs of any diameter, significantly lower compared with ILM peeling. Evidence on outcomes of vitrectomy without ILM peeling for small MHs is poor. Tadayoni et al. (Tadayoni et al., 2006) reported an 83% closure rate for no‐ILM peeling vitrectomy in MHs of any diameter. This rate decreased to 73% when considering MHs ≥400 μm. However, no‐ILM peeling technique yielded a 100% closure rate in <400 μm MHs, comparable with ILM peeling.^14^ Tognetto et al. (Tognetto et al., 2006) reported an 89% closure rate in <400 μm MHs. To date, no study has specifically investigated outcomes of no‐ILM peeling vitrectomy in MHs ≤250 μm.

The most recent classification of idiopathic MH proposed by the IVMTS defined as small holes those with a ≤ 250 μm diameter (Duker et al., 2013). This cut‐off was chosen by the IVMTS group because trials on pharmacologic vitreolysis showed that such a treatment was effective in ≤250 μm MHs, with a closure rate as high as 58%. MH closure was achieved thanks to the resolution of a VMT, without performing a vitrectomy (Haller et al., 2015; Stalmans et al., 2012). Diffusion of enzymatic vitreolysis has been limited because of the cost of ocriplasmin and the risk of sight‐threatening complications (Haynes et al., 2017).

The efficacy of pneumatic vitreolysis was also investigated in small MHs. Chan et al. (Chan et al., 2017) reported a 67% closure rate in ≤300 μm MHs treated with pneumatic vitreolysis. In all cases, a VMT traction was present and pneumatic vitreolysis led to a release of VMT in 86% of cases. The key mechanism to achieve anatomical success was the induction of posterior vitreous detachment, probably secondary to gas‐induced vitreous liquefaction (Chan et al., 2017). Subsequent VMT release and gas tamponade are supposed to promote hole closure. However, a recent clinical trial on pneumatic vitreolysis raised safety concerns due to retinal tears and retinal detachments, which led to an early termination of the trial (Chan et al., 2021).

Vitrectomy without ILM peeling ensures posterior vitreous detachment and VMT release, providing the same benefits as vitreolysis. Additionally, risks related to both enzymatic and pneumatic vitreolysis are avoided. Our report showed a closure rate of 85% after no‐peeling vitrectomy for small MHs. The rate of hole closure increased to 96% in the subgroup of eyes with MHs associated with VMT, which is similar to the 98% closure rate yielded by the ILM peeling technique. The multiple regression analysis confirmed that the presence of VMT was associated with a greater chance of hole closure in the no‐peeling group. These findings are in support of a vitrectomy without ILM peeling for small MHs associated with VMT. Vitreomacular traction is more likely in holes with a smaller diameter (Forsaa et al., 2018). It seems that the antero‐posterior traction component given by the VMT predominates in the pathogenesis of smaller holes, whereas the tangential traction component given by the ERM has a greater influence in larger holes.

Our multiple regression analysis showed that the presence of ERM was associated with a lower closure rate in eyes treated with ILM peeling. All this suggests that the aid of peeling is essential for the closure of larger holes that are more frequently associated with ERM, but it is less important in small holes, especially when associated with VMT.

Looking at the functional and OCT anatomical outcomes of closed MHs, results were better in the no‐peeling group. Mean postoperative BCVA gain was better at 6 and 12 months if peeling was not performed. This result could be due to damage induced by peeling and direct contact between retinal layers and surgical forceps, such as SANFL and DONFL previously investigated by other authors (Ehrhardt et al., 2023; Ito et al., 2005; Scupola et al., 2018). Not surprisingly, DONFL rate was higher in the ILM peeling group compared with the no‐peeling group (55% versus 9%, respectively). Furthermore, other aspects that could explain worse functional outcomes after ILM peeling include chromophore‐related or dye‐related toxicity, phototoxic damage, alteration of retinal nerve fibre layer thickness, attenuation of ganglion cell complex and shortening of papillofoveal distance (Almeida et al., 2015; Gelman et al., 2015; Ishida et al., 2023; Pradhan et al., 2022). Murphy et al. (Murphy et al., 2020) demonstrated better visual results after a foveal sparing ILM peeling. Preserving a rim of unpeeled ILM around idiopathic MHs could reduce Müller cell trauma, which, in turn, could improve functional and anatomical outcomes (Azuma et al., 2021).

When ILM was not peeled, the MH closure morphology was more similar to that of a spontaneous closure without surgical influences. The U‐shaped closure morphology seems to be the result of a regular regeneration of the fovea, with a centripetal displacement of the photoreceptor bodies that concentrates in the foveolar area and a concentric contraction of Müller cells in the outer plexiform layer (Bringmann et al., 2022; Sahoo et al., 2023). In addition to this, the lower rate of integrity of the external retinal layers (ELM and EZ) in the peeling group could be related to a higher presence of glial scar tissue in the foveola after surgical closure. It has been hypothesized that the glial scar is more likely to be persistent when integrity of ELM is not restored and that hypertrophied Müller cells in this scar tissue may impede regular regeneration of the outer nuclear layer and photoreceptor layer with a poorer visual outcome (Kitao et al., 2019).

Overall, rates of adverse events were comparable between ILM peeling and no‐peeling techniques with no safety concerns. Eyes with failed primary surgery achieved 100% closure rate after a second surgery.

The main limitation of this report is the imbalance in sample size between the peeling group and the no‐peeling group. The ILM peeling group was more than 10 times larger than the no‐peeling group, which significantly limits the statistical power of comparisons between the two groups. While this reflects real‐world clinical practice, it restricts the strength of our findings. The larger sample size in the peeling group aligns with the current preferences of surgeons in the surgical approach to small macular holes (MHs). However, we acknowledge that this imbalance may have impacted our conclusions and could potentially mask smaller differences in outcomes between the groups. A more balanced sample size would have provided greater clarity and strength in statistical comparisons, but the nature of a retrospective real‐life study means sample sizes cannot be predefined. A second significant limitation of the study was the retrospective design. This could have introduced some bias and reduced the preciseness of our estimates. Furthermore, the retrospective design did not allow to have data on an early follow‐up in eyes receiving air tamponade: a follow‐up earlier than 1‐month could have provided useful insights on early hole closure and visual recovery. Similarly, no data could have been extracted on symptom duration: it would have been interesting to investigate the influence of this clinical variable on our outcomes. However, small FTMHs are likely to have a short symptom duration compared with larger ones. Nonetheless, strict eligibility criteria were applied and a systematic protocol for data collection was adopted in order to reduce risk of bias. Additionally, the long‐term outcomes of both surgical techniques remain unclear. Our study was not designed to assess the long‐term durability of the benefits observed or potential late complications. Therefore, longer‐term follow‐up studies are necessary to evaluate the persistence of these benefits and to identify any delayed complications that might arise from either technique.

In conclusion, this report highlights that ILM peeling achieves higher closure rates for small idiopathic MHs. However, our results suggest that vitrectomy without peeling may be a viable option when a VMT is present. In such cases, the closure rates are similar to those observed with peeling, while both functional and anatomical outcomes tend to be more favourable, making the no‐peeling approach potentially preferable. These findings could inform surgical decisions in clinical practice, suggesting that a more individualized approach, tailored to specific patient characteristics, may be beneficial. However, further research is required to establish whether this strategy consistently optimizes both anatomical and functional outcomes and to better define its role in guiding surgical interventions.

## FUNDING INFORMATION

None.

## CONFLICT OF INTEREST STATEMENT

No conflicting relationship exists for any author.

## APPENDIX A

### A.1

**SMALL Study Group**: Tommaso Micelli Ferrari, Massimo Lorusso, Vito Primavera, Gianluigi Giuliani, Cesare Mariotti, Marco Lupidi, Luca Ventre, Giulia Pintore, Lorenzo Motta, Matteo Ripa, Francesco Boscia, Giacomo Boscia, Mario R Romano, Mariantonia Ferrara, Kacerik Miroslav, Daniele Marchina, Barbara Parolini, Enrico Peiretti, Viola Marchiori, Roberto Dell'Omo, Marzia Affatato, Teresio Avitabile, Andrea Russo, Antonio Longo, Vincenzo Scorcia, Adriano Carnevali, Rodolfo Mastropasqua, Lisa Toto, Agostino Salvatore Vaiano, Riccardo Merli, Marco Mura, Marco Pellegrini, Fabrizio Giansanti, Cristina Nicolosi, Matteo Badino, Nicola Pallozzi Lavorante, Maria T. Sandinha, Francesco Maria D'Alterio, Mario Damiano Toro, Robert Rejdak, Paolo Chelazzi, Claudia Azzolini, Francesco Viola, Caterina Donà, Matteo Giuseppe Cereda, Marco Casaluci, Marco Codenotti, Lorenzo Iuliano, Grazia Pertile, Daniele Sindaco, Stefano De Cillà, Andrea Muraca, Vincenza M.E. Bonfiglio, Maria Vadalà, Alberto La Mantia, Viviana Randazzo, Tito Fiore, Gianluigi Tosi, Rino Frisina, Chiara Angeli, Marco Coassin, Mariateresa Laborante, Tommaso Rossi, Pamela Cosimi, Stanislao Rizzo, Matteo Mario Carlà, Magda Gharbiya, Giuseppe Maria Albanese, Luigi Caretti, Edoardo Angelini, Gian Marco Tosi, Tommaso Bacci, David H Steel, Nikolaos Dervenis, Iordanis Vagiakis, Daniele Tognetto, Marco Rocco Pastore, Francesco Faraldi, Carlo Alessandro Lavia, Paolo Lanzetta, Daniele Veritti, Leopoldo Rubinato, Paolo Radice, Andrea Govetto.
